# Illuminating the path to more equitable access to urban parks

**DOI:** 10.1038/s41598-025-94110-1

**Published:** 2025-03-20

**Authors:** Kaihan Zhang, Wen-Long Shang, Jonas De Vos, Yuerong Zhang, Mengqiu Cao

**Affiliations:** 1 Bartlett School of Planning, University College London, London, UK; 2 Cho Chun Shik Graduate School of Mobility, Korea Advanced Institute of Science and Technology, Daejeon, South Korea; 3 Centre for Transport Studies, Imperial College London, London, UK; 4 Bartlett School of Environment, Energy and Resources, University College London, London, UK

**Keywords:** Urban park accessibility, Multimodal travel, Green justice, Streetscape quality, GeoAI, Psychology and behaviour, Socioeconomic scenarios, Sustainability

## Abstract

Parks are a crucial element of urban landscapes. Understanding the role of the built environment in terms of park accessibility is vital for promoting travel equity and shaping public policies to improve access to urban parks. This study employs a geospatial artificial intelligence (GeoAI) approach to examine the impact of the built environment on park accessibility at both macro and micro levels. We find that accessibility to urban parks varies significantly across different travel modes, with walking being the least equitable due to its most clustered distribution pattern. While the macro-level built environment provides substantial explanatory power for accessibility across travel modes, our GeoAI-based analysis offers a more nuanced understanding of streetscape qualities, by closely reflecting a more realistic human perception of the built environment. These qualities - street network interconnectivity, safety, openness, spaciousness, greenery, and distinctive signage - significantly correlate with enhanced park accessibility across all travel modes, thereby complementing macro-level factors. Ultimately, this study suggests that placing emphasis on multimodal travel can enhance travel equity, thus providing the relevant authorities and policymakers with cost-effective strategies for improving park accessibility.

**Supplementary Information:**

The online version contains supplementary material available at 10.1038/s41598-025-94110-1.

## Introduction

Urban parks are essential for outdoor recreation, social interaction, and connecting with nature^1^, offering numerous environmental, social, and psychological benefits to individuals and communities in urban areas^2^. The quality of life for urban residents is closely tied to the accessibility of these natural spaces^3^. However, these benefits can only be realised when parks are accessible and usable by city residents. Therefore, ensuring the equitable distribution and accessibility of urban parks is crucial for promoting equal, diverse and inclusive (EDI) urban development.

Accessibility refers to the ability to reach spatially distributed opportunities, such as parks^4^, and is a key factor influencing the use of urban green spaces^5^. Studies have shown that accessibility, often measured by proximity, positively affects park usage^6^. Extending our understanding of the factors influencing urban park accessibility (UPA) offers valuable insights for planners and the relevant authorities as well as potential ways of promoting park use. This would ensure that residents are able to fully benefit from urban parks while addressing the issue of spatial equity through fairer access^7^.

As accessibility is widely recognised as a key determinant of park visits, an expanding body of research has examined its associations with other potential multidimensional factors. From a macro perspective, several key elements that influence UPA have been identified, including transport infrastructure, street network design, and land use types. European and Canadian studies have emphasised the significance of public transport and urban transit stations in relation to park accessibility^8,9^, while other research has highlighted the benefits of well-connected street networks for accessing services and physical activity by offering shorter pathways and seamless transitions between diverse route options^10–12^, particularly walking^13^. Street network design has also increasingly come to incorporate spatial syntax^14^, which applies graph theory to street networks, offering valuable insights into evaluating park accessibility from a topological perspective^15^. This approach provides useful guidance for designing more efficient and accessible urban spaces. In addition, there is broad agreement in the literature that land use, particularly its type and distribution, significantly affects the accessibility of urban parks and green spaces^16–18^. However, the extent of this influence and whether it is positive or negative in nature remains uncertain. Current literature primarily focuses on the singular perspective of the aforementioned macro-scale built environment, without adequately addressing the micro-scale, particularly human impressions of streetscape qualities. We hypothesise that these perceived streetscape features can complement conventional built environment factors to better inform park accessibility. This view is supported by studies suggesting that geographic quantification alone is insufficient to gain deeper insights into park accessibility, and that individual perceptions of parks may play an increasingly influential role^5^. The dynamics of how these factors impact on UPA, both individually and collectively, remain largely unexplored. In addition, these studies often overlook multimodal travel, limiting our understanding of accessibility through various modes of transport^19,20^. Hence, a comprehensive framework integrating both the macro-scale built environment and micro-scale streetscape qualities within the wider scope of multimodal travel options is urgently needed.

Despite the importance of perceived streetscape quality in urban planning, particularly for physical activity and well-being^21^ being acknowledged, its role in relation to park accessibility has received less attention. Traditional assessments of streetscape quality often rely on subjective methods, such as surveys and interviews^22–24^, which may not accurately represent realistic eye-level perceptions due to individual variations. GeoAI, which integrates artificial intelligence with spatial analysis, has gained prominence in urban analytics due to its ability to integrate spatial concepts and reasoning into advanced AI models, particularly deep learning, in order to resolve diverse geographical challenges. These challenges include land use classification, spatial object detection from remote sensing images^25^, and place perception using geo-tagged imagery^26^. Of these applications, place perception is particularly important for addressing urban-related issues. Notable efforts have been made to leverage human environmental perceptions based on geo-tagged imagery to explain perceptions of safety^27,28^, the occurrence of violent crime^29^, and to reveal large-scale social-demographic differences^30^. The technical approaches used in this field heavily rely on semantic segmentation techniques being applied to street view imagery, enabling the extraction of fine-grained street-level features. Beyond this, GeoAI-based methods are able to rapidly cover larger areas, such as entire cities, while reducing bias and offering a more cost-effective and time-efficient solution compared to traditional approaches^27^. Despite its advantages, the association between streetscape quality, as measured by GeoAI, and urban park accessibility, remains underexplored. This approach provides a deeper, additional micro-level understanding of streetscape quality, complementing macro-level analyses and offering a comprehensive framework with which to better inform urban park accessibility. This study aims to unveil the complex relationships between the multi-scale built environment and park accessibility in a multimodal travel context.

To address this research area, we advocate a GeoAI-based, multi-scale framework that offers promising insights for research on park accessibility in a multimodal travel context. Our nexus approach integrates urban mobility, deep learning, and urban planning, thereby shedding light on the relationship in question.

## Data and methods

### Study area

**Fig. 1:**
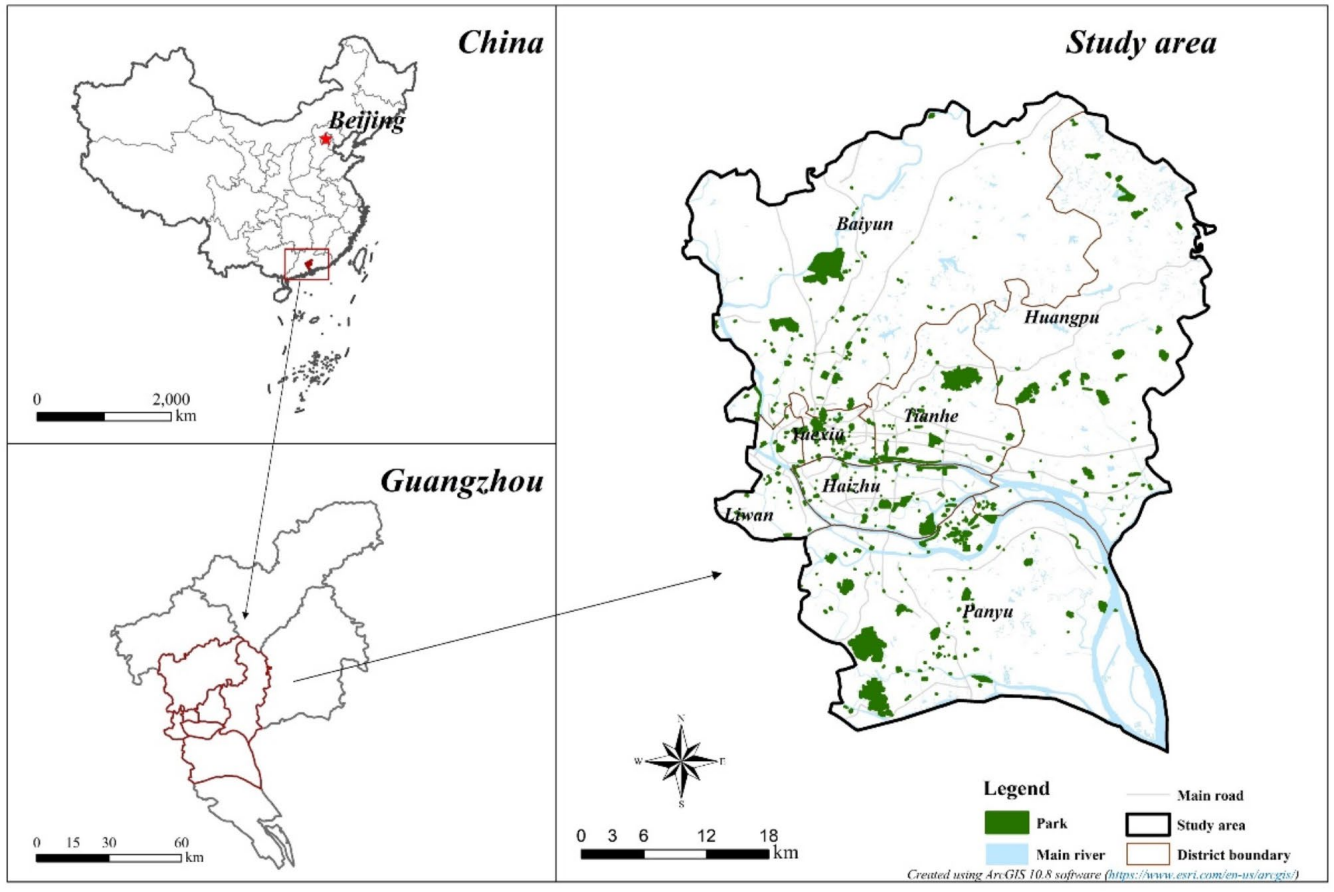
Map of study area. (Maps created using ArcGIS 10.8, https://www.esri.com/en-us/arcgis)

This study focused on seven core districts of Guangzhou, China (population: 18.68 million), namely: Panyu, Haizhu, Huangpu, Liwan, Tianhe, Yuexiu, and Baiyun (Fig. 1). These districts are integral to Guangzhou’s strategic urban plans, including the “2035 Global Liveable Huacheng”, “Vibrant Park City” and “Pearl River Delta National Forest City Group”^31^. According to the Guangzhou Statistics Bureau^32^, these areas contain 70% of the city’s urban parks and 75% of its population. Equipped with a well-developed street network and transport infrastructure, they serve as Guangzhou’s economic, cultural, residential, and political centres, providing an opportunity to evaluate park accessibility in the central urban areas of the province. Guangzhou’s rapid urbanisation, dense population and ambitious planning make it a representative case for rapidly urbanising cities worldwide that face challenges related to inequitable access to urban parks.

### Data collection and processing

**Fig. 2:**
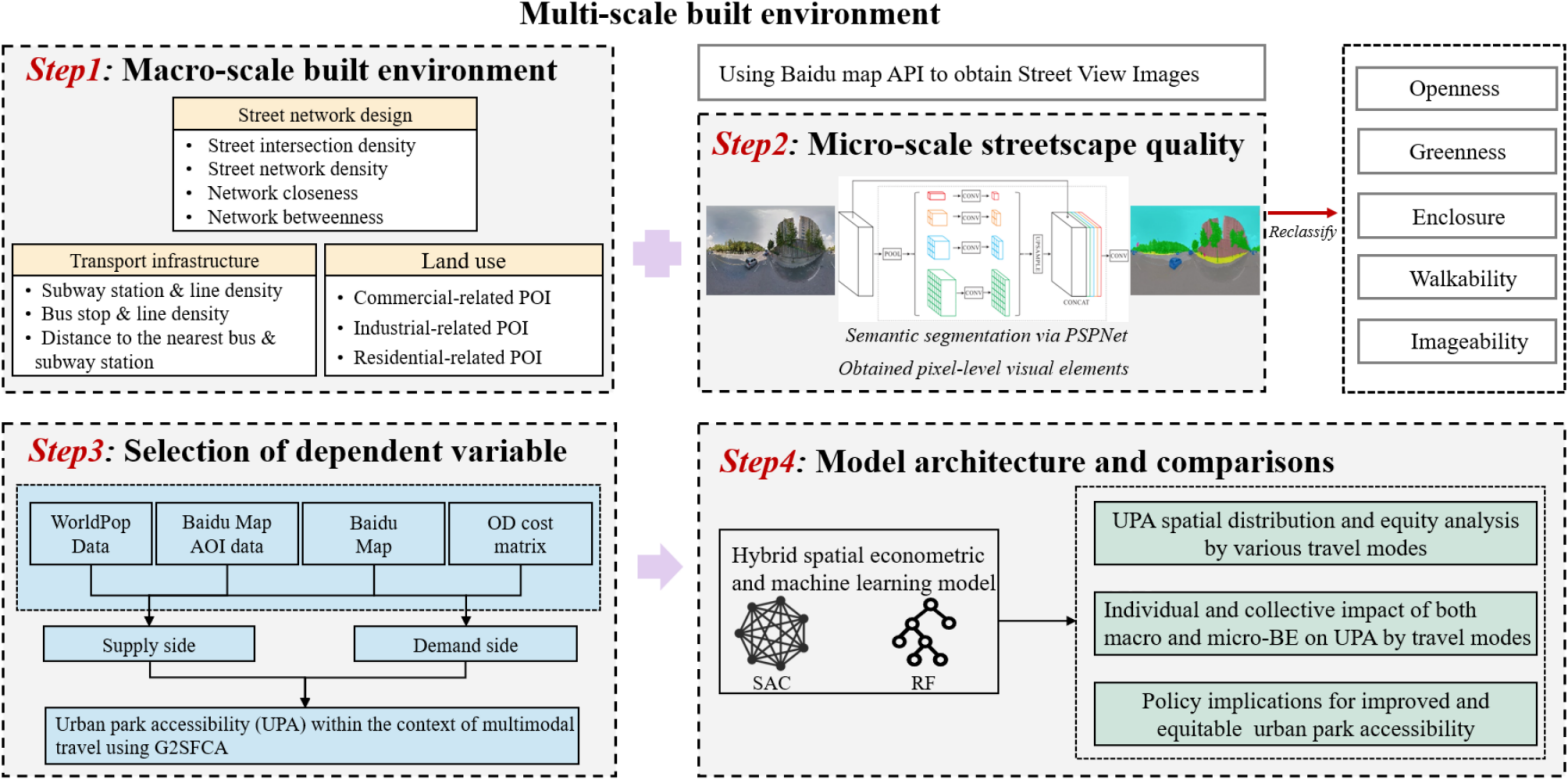
Research roadmap.

Multi-source datasets from various relevant sources were compiled to assess park accessibility across different travel modes and categorised into three main types: street view images (SVI), demographic characteristics, and urban environmental attribute data. Notably, data on Guangzhou’s administrative decisions were obtained from the Guangzhou Public Data Open Platform in 2023^33^. Urban park Area of Interest (AOI) data were extracted from Baidu Maps via a Python script using the map API. This resulted in a total of 498 parks being identified in the study area. Different types of scenic spots within these AOIs were validated for accuracy against GF-1 satellite imagery. Transport infrastructure and road network data were sourced from OpenStreetMap and refined using Baidu Maps to ensure accuracy and relevance. SVI data were collected via a Python script interfacing with the Baidu SVI static API, which captured pedestrian eye-level images from four directions at 50-metre intervals along the road network. This dense sampling facilitated comprehensive coverage and detail. The images underwent semantic segmentation via image segmentation algorithms, using the PSPNet model^34^, trained on the CityScapes^35^ 50-city autopilot dataset for pixel-level object identification. This process efficiently segregated pavements, trees, buildings, and other elements (refer to Fig. 2), excluding transient visual elements such as cars, bicycles, and motorcycles, to maintain consistency with existing research methodologies^36^. Five visual perception indices - greenness, openness, enclosure, walkability and imageability - were computed from the segmented data, following prior methodologies^36–38^. These indices, derived from specific pixel proportions of visual elements, are detailed in the Supplementary material (Table 1), offering a nuanced understanding of streetscape quality.

### Methodological framework

The first section of the research roadmap integrates road network data, population distribution data, and urban park AOI data. It employs a Gaussian-modified Two-Step Floating Catchment Area (G2SFCA) method^39^ to analyse the accessibility of urban parks via walking, cycling and driving. A description of the G2SFCA is provided in Text S2 in the Supplementary material. Local Moran’s *I* statistics are then applied to identify the spatial patterns and equity of park accessibility. The second section summarises key factors influencing urban park accessibility, as identified in existing literature, with a primary focus on the macro-level built environment and the inclusion of streetscape quality features through a GeoAI approach. In the third section, following collinearity testing and standardisation, random forest (RF) and spatial econometric modelling, namely spatial autocorrelation models (SAC), are employed to examine the complex relationships between various factors and UPA across different travel modes. Finally, the model results are discussed, with a view to providing recommendations for enhancing park accessibility.

### Spatial autocorrelation analysis

Determining the presence of spatial autocorrelation between variables is a prerequisite for performing spatial econometric analysis. Some of the most prevalent approaches used to do so include Geary’s C index, Geti’s G index, and Moran’s *I* index, with the latter being the most frequently employed. Moran’s *I* measures the extent of attribute value similarity across spatially adjacent or neighbouring regions, thus serving as a tool for examining the spatial correlation between variables. The formula is specified as follows:

1 $$I=\frac{{\sum\limits_{{{\text{i}}=1}}^{{\text{n}}} {\sum\limits_{{{\text{j}}=1}}^{{\text{n}}} {{w_{ij}}} } ({x_i} - \bar {x})({x_j} - \bar {x})}}{{{S^2}\sum\limits_{{i=1}}^{n} {\sum\limits_{{j=1}}^{n} {{w_{ij}}} } }}$$

Within this framework, $\:{S}^{2}=\frac{1}{n}\sum\:_{i=1}^{n}{\left({x}_{i}- \mathop{x}\limits^\leftharpoonup \right)}^{2}, \mathop{x}\limits^\leftharpoonup =\frac{1}{n}\sum\:_{i=1}^{n}{x}_{i}$ denotes the observation of the $\:{i}{th}$ region, $\:{S}^{2}$ is the sample variance, n is the total number of regions, and $\:{w}_{ij}$ is the $\:i$, $\:{j}{th}$ element of the spatial weight matrix. Moran’s *I* index takes values in the range [-1,1].

A positive Moran’s *I* index indicates positive spatial autocorrelation, with values close to 1 showing strong clustering of similar values - high-value regions near other high-value regions, and low-value areas near low-value regions. A Moran’s I index of 0 implies no spatial correlation, with high and low values randomly distributed. A negative index denotes negative spatial autocorrelation, with values near −1 indicating stark contrasts between neighbouring areas, where high-value regions border low-value regions.

### Spatial econometric model selection

Moran’s *I* index identifies the presence and strength of spatial autocorrelation without specifying the appropriate spatial model. Hence, the LM test is essential for selecting the correct spatial interaction model. Specifically, the LM-error test, introduced by Burridge in 1980^40^, and the LM-lag test, proposed by Anselin (1988)^41^, are crucial in this respect. The LM-error and LM-lag tests were enhanced through the introduction of their robust counterparts, the robust LM-error and robust LM-lag tests^42^. The equations for these tests are presented below:

2 $$RobustLM - Error={\left( {\frac{{e^{\prime}Wy}}{{{s^2}}} - \frac{{T{R^{ - 1}}e^{\prime}We}}{{{s^2}}}} \right)^2}/(T - {T^2}{R^{ - 1}})$$

3 $$Robust{\text{ LM}} - L{\text{ag}}={\left( {\frac{{{{e^{\prime}}}Wy}}{{{s^2}}} - \frac{{e^{\prime}We}}{{{s^2}}}} \right)^2}/(R - T)$$

$\:{e}^{{\prime\:}}Wy$ is the spatial lag of the residuals, with $\:e$ being the vector of residuals from an OLS regression, $\:W$ is the spatial weights matrix, and $\:y$ is the dependent variable. $\:{s}^{2}$ is the squared standard error of the residuals from the OLS regression. $\:T{R}^{-1}{e}^{{\prime\:}}We$ is an adjustment term that accounts for the influence of the independent variables on the spatial error process. $\:T$ is the trace of the spatial weight matrix, indicating spatial dependence levels. $\:T$ pertains to the Moran’s *I* statistic matrix for spatial autocorrelation.

### Feature importance ranking

The Random Forest algorithm, a widely recognised machine learning method, is known for its efficiency in evaluating the importance of multiple variables when predicting outcomes. It was selected for its superior performance, as it achieved the lowest Root Mean Squared Error (RMSE) and Mean Absolute Error (MAE) on this dataset, outperforming other commonly used machine learning models across three travel modes. Feature importance in RF is determined by the reduction in mean squared error (MSE) at each split. Specifically, the importance of a feature is calculated by summing the reductions in the MSE at splits that involve the feature, and then averaging these values across all trees in the forest. In this study, the dataset was divided into training and testing sets using a 70:30 ratio. RF can be instrumental in filtering out irrelevant variables and identifying the relative importance of each independent variable. Subsequently, feature importance scores were calculated using Scikit-learn in Python.

## Results

### UPA Spatial distribution pattern and equity analysis

In this study, we designated a 500 m threshold grid to capture detailed interactions between park accessibility and spatial structure. This decision was based on a spatial autocorrelation test using Moran’s *I* to identify an appropriate distance threshold^43^. This *spatial threshold* defines the spatial weight matrix and the spatial relationships between park accessibility values, which is essential for accurate spatial interaction modelling^44^. A 15-minute duration was adopted as the critical time threshold, reflecting the concept of the ‘15-minute city’, which posits that essential services should be accessible within a 15-min walk or cycle ride. This concept is fundamental in shaping the structure of community life^45^. The spatial distribution patterns of UPA across different travel modes are shown below.

**Fig. 3:**
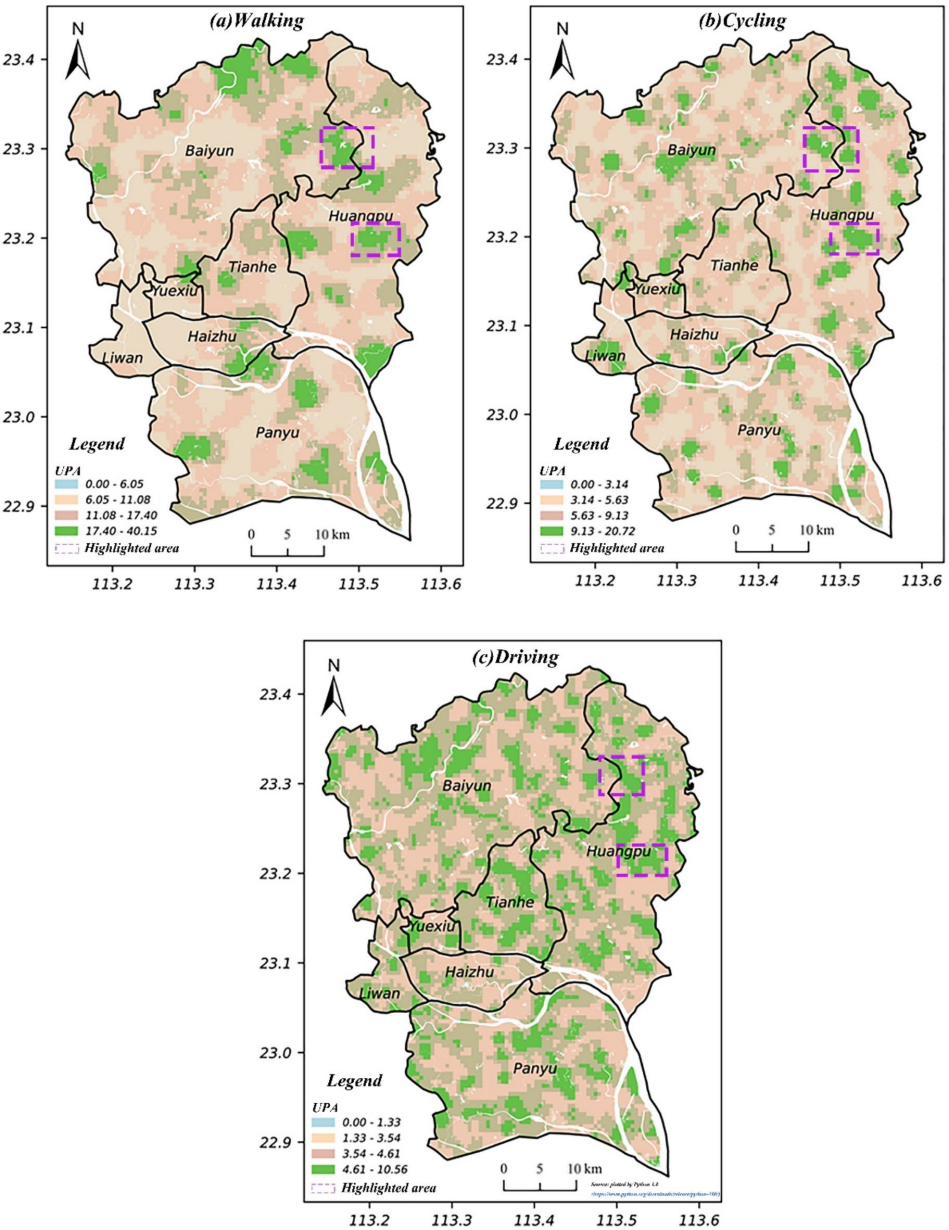
UPA via the three travel modes. (Maps plotted using Python 3.8, https://www.python.org/downloads/release/python-380)

Each grid’s UPA value was categorised into four distinct types using the Jenks Natural Breaks Classification method. This method identifies breakpoints between groups by minimising intra-group variation. A higher UPA value for a grid indicates better park accessibility. Overall, UPA distribution patterns differed between the three modes, especially in the high accessibility zone. In the case of walking, grids with high accessibility are concentrated in a few areas, while the majority have a lower UPA. The high accessibility zones are most prevalent in relation to driving, followed by cycling and then walking. This indicates that urban parks are, not surprisingly, most accessible by car. This also suggests a more clustered park accessibility distribution for walking and a more dispersed distribution for the other two modes. Thus, the UPA pattern for walking has a more inequitable distribution. Typically, a 15-minute cycling or driving range covers a larger distance area than walking. However, this does not necessarily mean that cycling and driving have an absolutely higher UPA than walking. As the 15-minute range increases with stable park provision, it includes more of the population, leading to a decline in park accessibility. The highest accessibility values underscore this observation: 40.15 for walking, 20.72 for cycling, and 10.56 for driving. In essence, the magnitude of park accessibility depends on the size and distribution of parks and population.

The areas marked by the purple boxes in Fig. 3 typically have higher accessibility values across all three modes. Areas with lower accessibility often relate to walking, the most common and frequent travel choice among residents^46^. Two scenarios can potentially explain the phenomenon of low accessibility areas. On one hand, in suburban areas like northwest Baiyun, Panyu, and east Huangpu, which are relatively distant from the city centre, park provision is insufficient (see Fig. 1). On the other hand, in areas such as central Liwan, Yuexiu, and west Haizhu, distinguished by economic prosperity and high population density, there is ample provision of urban parks. However, the higher population also elevates demand, potentially reducing park accessibility. In the first scenario, such areas can offset the reduced accessibility of cycling (see Fig. 3b). For areas in the second scenario, the extent to which accessibility can be improved across all modes is limited. An exception is southern Liwan, where cycling has notably boosted park accessibility over walking. This can be attributed to the prevalence of roads designed for cyclists in these regions^47^.

In general, park accessibility varies spatially based on travel mode, revealing an uneven distribution across all modes, with walking displaying the greatest inequity in relation to accessing urban parks. Areas with low park accessibility typically include suburban regions with inadequate park provision, and densely populated city centres with a high demand for leisure activities. In areas where walking accessibility is low, cycling can serve as an alternative to enhance access to parks.

**Fig. 4:**
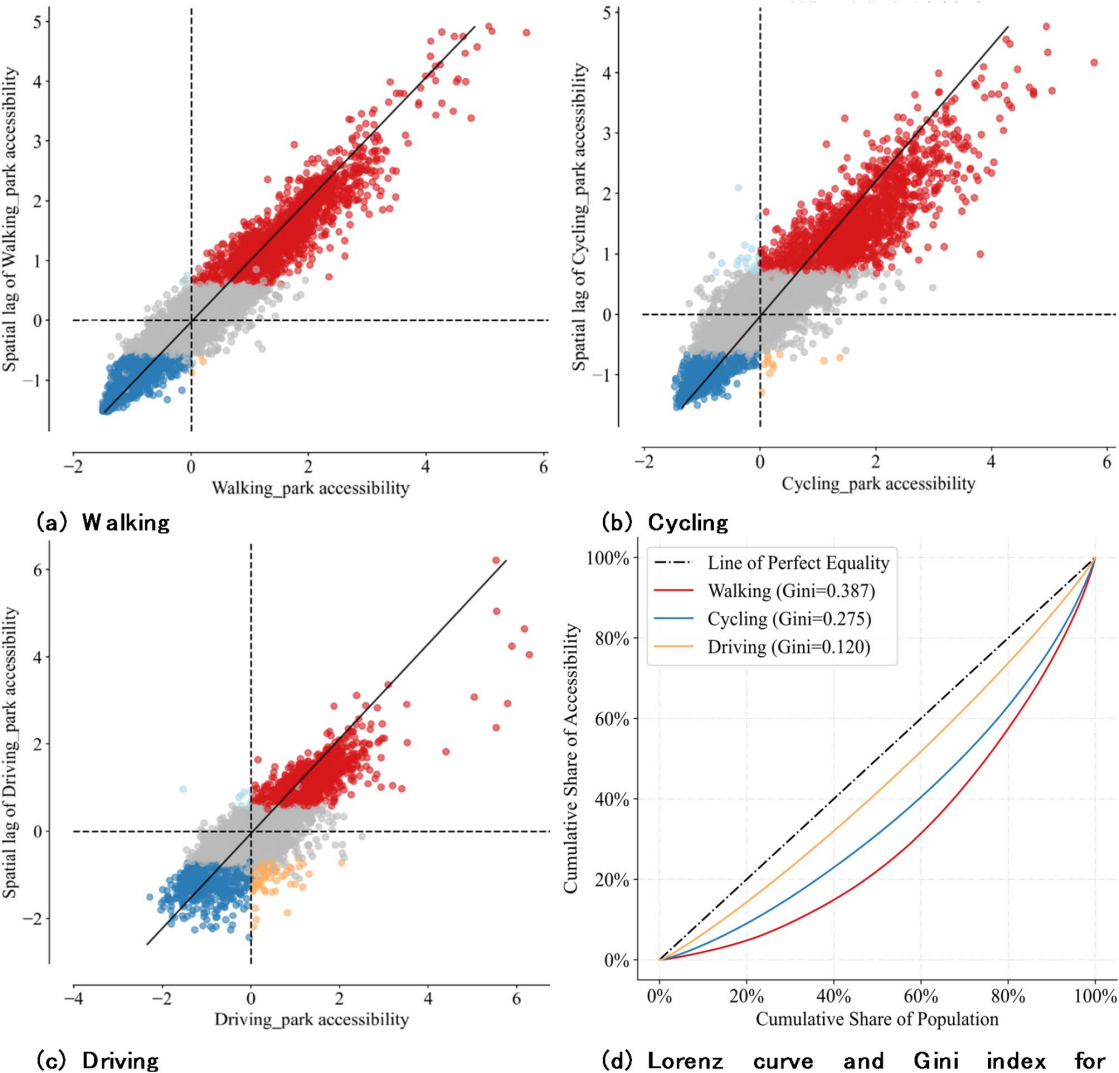
Moran’s *I* scatter plot (**a**), (**b**), (**c**) and equity assessment (**d**) by travel modes.

The preceding analysis revealed a pronounced spatial clustering effect for each travel mode, as illustrated by the Local Moran’s *I* scatter plot. Walking exhibited a Global Moran’s *I* value of 0.864. Similar clustering patterns were observed for cycling and driving, with Global Moran’s *I* values of 0.746 and 0.682, respectively (see Supplementary Material, Table 2). These significant Global Moran’s *I* values are consistent with the distribution patterns in the Local Moran’s *I* scatter plot, where most areas fall into high-high or low-low clusters, represented by red and blue, respectively in Fig. 4a, b and c. This indicates that regions with high accessibility are often surrounded by other high-accessibility areas, while regions with low accessibility are similarly surrounded by low-accessibility areas.

To further quantify and evaluate the inequity in park accessibility across travel modes, the Gini index was calculated using the formula below, and the Lorenz Curve was plotted in Fig. 4d.

4 $$G=1 - \sum\limits_{{i=1}}^{n} {\left( {{A_{i+1}}+{A_i}} \right)} \left( {{p_{i+1}} - {p_i}} \right)$$

Where G indicates the Gini coefficient; n represents the total number of grids (spatial units); ${p_i}$ is defined as the cumulative share of population up to rank *i* and${A_i}$ is the cumulative share of accessibility up to rank *i*, with ${p_0}={A_0}=0$and ${p_n}={A_n}=1$.

The extent to which the Lorenz Curve deviates from the diagonal, along with the corresponding Gini index value, reflects the level of inequity. More specifically, a Gini index of greater than 0.2 is deemed as moderate inequity^48,49^. This implies that active travel modes, such as walking and cycling - characterised by slower speeds - are more susceptible to inadequate urban park accessibility. In contrast, driving benefits from higher speeds, enabling greater area coverage and resulting in lower inequity levels. Based on these findings, it is reasonable to infer that this inequity may be even more pronounced among individuals with limited car ownership affordability. In other words, those who primarily rely on active travel modes are more likely to become transport-induced green justice disadvantaged groups.

### Relationships between variables and urban park accessibility

The Moran’s *I* results indicated spatial autocorrelation across all travel modes, prompting the use of a spatial effects model. A Pearson correlation analysis was conducted, and only variables with a statistically significant correlation (*p* < 0.1) were included in further modelling, to avoid complexity and overfitting^50^. The robust Lagrange Multiplier (LM) test identified spatial lag and error effects (see Supplementary Material, Table 2), leading to the adoption of the SAC model, which combines the Spatial Lag Model (SLM) and the Spatial Error Model (SEM). The subsequent analysis focuses on the results produced by the SAC model using the spatial weight matrix W, measured by the queen contiguity method, and random forest feature importance within the multimodal travel context.

**Table 1:** Results of the SAC models by travel modes.

Aspect	Variables	SAC model (walking)			SAC model (cycling)			SAC model (driving)		
		Coef.	Sig.	Std. error	Coef.	Sig.	Std. error	Coef.	Sig.	Std. error
Macro-scale Built environment. (MBE)	Intercept	-0.077	/	0.119	-0.025	/	0.048	-0.022	/	0.062
	Subway station density	0.001	/	0.003		/		-0.002	/	0.004
	Bus stop density	-0.011	***	0.004	-0.009	/	0.006	-0.023	***	0.006
	Subway line density	-0.003	/	0.013	-0.013	/	0.017	-0.024	/	0.017
	Bus line density	-0.053	***	0.015		/		0.010	/	0.020
	Distance2bus	-0.01	/	0.009	-0.022	*	0.013	-0.004	/	0.013
	Distance2sub	0.023	***	0.007	0.028	/	0.041	-0.091	*	0.048
	Closeness	0.023	***	0.008	0.050	***	0.011	0.090	***	0.011
	Betweenness	-0.004	/	0.005	-0.009	/	0.007	-0.002	/	0.006
	Road network density	0.031	***	0.009	0.035	***	0.013	0.020	/	0.012
	Intersection ratio	-0.004	/	0.004	-0.003	/	0.006	-0.009	/	0.006
	Commercial	0.016	***	0.006	0.007	/	0.008	0.033	***	0.007
	Residential	0.003	/	0.006	-0.004	/	0.009	0.024	***	0.008
	Industrial	0.009	*	0.005		/		0.009	/	0.006
Micro-scale Streetscape quality (SQ)	Greenness	0.167	***	0.006	0.152	***	0.008	0.359	***	0.008
	Openness	0.293	***	0.005	0.285	***	0.007	0.625	***	0.007
	Enclosure	-0.004	/	0.004	0.004	/	0.005	0.001	/	0.005
	Walkability	0	/	0.004	0.005	/	0.005	0.010	*	0.005
	Imageability	0.067	***	0.005	0.095	***	0.007	0.198	***	0.007
	Pseudo R^2^	0.912			0.839			0.845		
	Wy	-0.1	***	0.023	0.335	***	0.027	0.153	***	0.02
	Lambda	0.97	***	0.003	0.912	***	0.008	0.933	***	0.005
	AIC	5998.5			11,102			10,817		
	AIC for lm	24,562			25,102			22,233		
	Log likelihood	-2977			-5531			-5386		
	Log likelihood lm/for OLS	-12,261			-12,532			-11,096		

No. observations: 9062; ***, **, and * ==> significance at 1%, 5%, 10% level; / indicates not significant.

### Effects of the macro-scale built environment

**Fig. 5:**
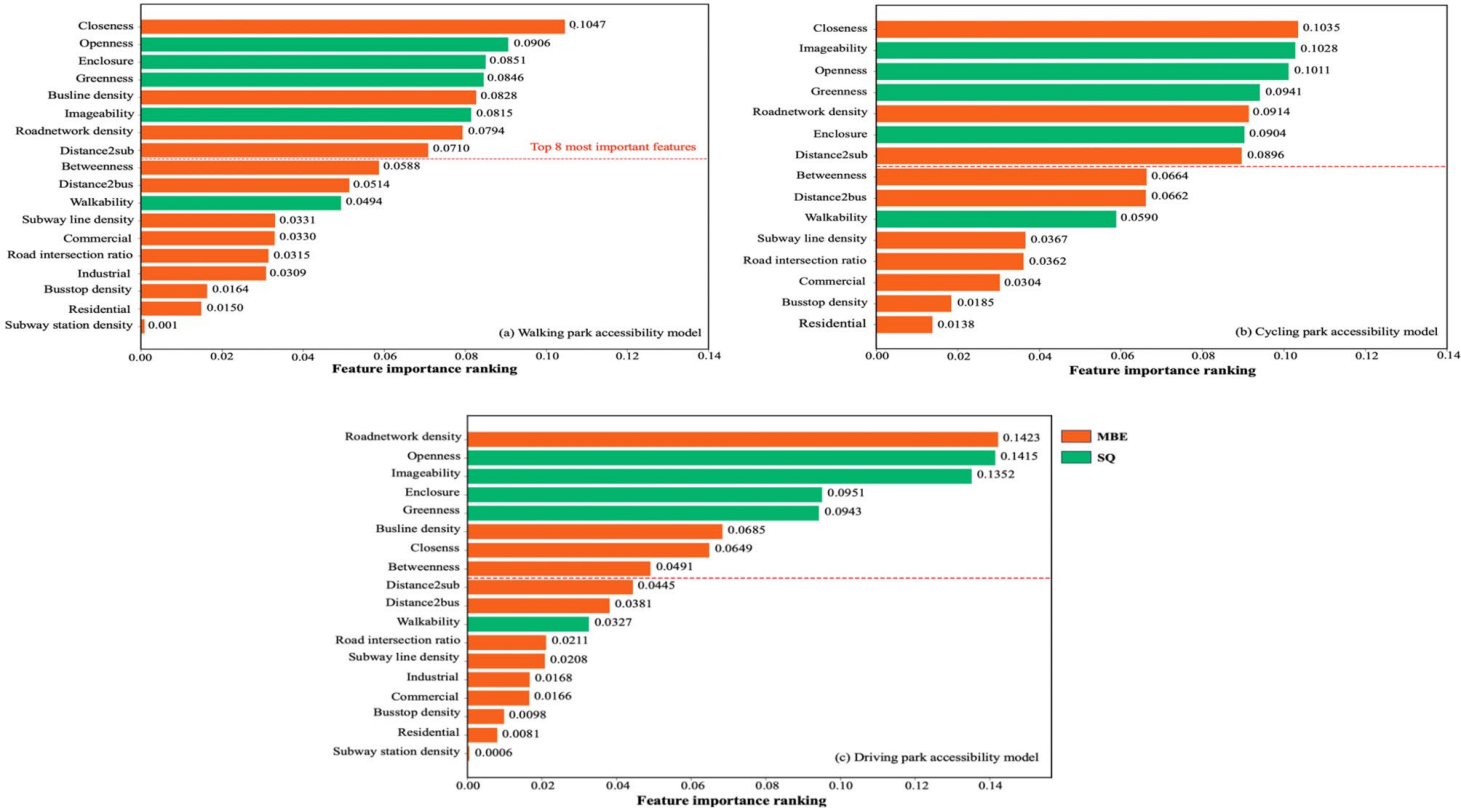
Random forest feature importance rankings of variables in relation to walking (**a**), cycling (**b**), and driving (**c**) park accessibility.

The transport infrastructure variables exhibit contrasting significance within the active travel modes. For instance, while bus stop and line density have a significant impact on walking UPA and are prominent in the random forest feature importance ranking results (see Fig. 5), they have no impact on cycling. Interestingly, ‘Distance2bus’ is negatively significant in relation to cycling UPA, while ‘Distance2sub’ is only significant in relation to walking UPA. This result suggests that ‘Distance2bus’ may marginally limit cycling accessibility to parks. One potential explanation for this is that bus trips may substitute certain cycling journeys within a specific distance, suggesting a competitive relationship between the two modes^51^. The ‘Distance2sub’ has a positive effect on park accessibility by walking, with proximity to a subway station contributing to a higher walking UPA. The observed phenomenon suggests the potential for integrating public transport in order to enhance park accessibility. These findings align with previous studies which have shown that park accessibility is influenced by proximity^52,53^. However, a contrasting study from Denmark found that distance was not a limiting factor for local residents, with most able to access green spaces within 300 metres^54^. These mixed results may be due to differences in geographical context.

From the perspective of street network design, a systematic review has highlighted the crucial role of street network design in park accessibility, although its impact across different travel modes remains unclear. Using spatial syntax, this study reveals a significant positive correlation between network closeness and urban park accessibility for all three travel modes (walking, cycling, and driving). Well-connected streets are associated with higher park accessibility, with closeness being the most influential factor for active travel (walking, cycling) and relatively less important for driving. For pedestrians, given their slower pace, closeness is vital as shorter paths directly enhance accessibility, implying a more convenient and well-connected route. However, for faster modes of travel such as driving, the ability to cover distances rapidly makes the immediate proximity of the shortest route less consequential. This suggests that, as travel speed increases, the relevance of street network attributes in affecting UPA decreases. Although spatial syntax is gaining traction in urban park research^55–57^, evidence linking street network design with park accessibility remains limited. Existing studies mainly show that interconnected street networks improve walkability and reduce travel time to green spaces^53^. Our finding that street network design positively influences park accessibility across all travel modes is novel. Most importantly, it was statistically significant in the regression model and ranked highest in terms of feature importance in the machine learning analysis. ‘Closeness’, particularly for walking and cycling scenarios, emerged as the most important variable, underscoring the role of street network connectivity in relation to park accessibility for active travellers.

Land use plays only a limited role in influencing park accessibility. The study found a positive correlation between commercial areas and UPA for driving, which contrasts with previous findings from a systematic review that suggested commercial facilities could hinder accessibility^53^. In the case of active travel, land use factors do not significantly affect cycling accessibility. However, a positive relationship between commercial land and park accessibility for walking suggests that denser commercial areas are associated with improved park access by walking, which contrasts with U.S. studies showing that parks in residential areas attract more users than those in commercial zones^58^. Despite prior doubts about the direct association between land use and park accessibility, this study confirms the association statistically. A plausible explanation for this is that denser commercial areas often stimulate walking, as well as driving investment in pedestrian infrastructure, such as pavements, crosswalks, and signals. These enhancements improve safety and convenience, potentially increasing access to parks and other amenities.

### Effects of micro-scale streetscape quality

As observed above, significant streetscape quality features are positively correlated with park accessibility. The machine learning analysis (Fig. 5) further reveals the ranking of these variables based on the magnitude and relative importance of their effects. The results indicate that a few micro-level streetscape determinants have greater explanatory power than most macro-level built environment factors across all travel modes. A detailed comparison by travel mode highlights that openness and greenness consistently rank among the top five factors, underscoring their crucial role in park accessibility. Regarding the estimated coefficients and their statistical significance (Table 1), the signs and magnitudes of these coefficients remain consistent across travel modes. Streetscape openness and greenness exert the highest and second-highest impacts, respectively, showing a strong positive correlation with urban park accessibility. This statistical finding aligns with the feature importance rankings.

These complementary findings indicate that spacious streets with greater sky visibility and street-level greenery may improve park accessibility. Greenness, measured from a micro perspective, emerges as a strong predictor of park accessibility. The higher feature importance ranking suggests that residents’ perceptions of street-level greenery better explain park accessibility, whereas previous studies found no significant association between overhead greenery, measured by NDVI, and park usage^59^. Interestingly, the attribute of ‘walkability’ often implies the suitability of a street for walking, with wider streets typically correlating with a stronger sense of pedestrian safety^60^. However, it did not significantly influence active travel and had a relatively low feature importance score. One possible explanation for this is that the components of walkability are derived from SVI calculations, which rely on views captured by cameras embedded in moving vehicles. The unique perspectives provided by these cameras, combined with the static nature of the images, may obscure some pavements, leading to a lower walkability score. This could also explain why the coefficients vary significantly across different travel modes. However, the coefficients of the ‘imageability’ variable were consistently positive across all three travel modes. Streets with high imageability values tend to be distinctive, easily recognisable, and aesthetically pleasing, which is conducive to park accessibility across all travel modes.

## Discussion

### The collective impact on park accessibility

Incorporating spatial effects provides further evidence that these models outperform OLS linear regression, as reflected in the fit statistics shown at the bottom of the table. The collective impact of macro-scale BE and micro-scale SQ variables provides greater explanatory power than either alone. For example, micro-scale SQ alone achieved a Pseudo R² value of 0.534 for UPA by walking, while the corresponding value for macro-scale BE was 0.848. Integrating both scales resulted in a Pseudo R² value of 0.912. This confirms that micro-scale SQ features complement macro-scale BE characteristics in explaining UPA. Previous studies have often focused solely on macro-scale BE^12,61^, neglecting a holistic, multi-scale analysis and overlooking the importance of micro-scale features. The tendency to overlook these features, which are essential for capturing immediate human impressions of the streetscape^50^, results in an inability to accurately reflect these perceptions. Ignoring them diminishes the relevance of findings for human-centred planning. Therefore, integrating both MBE and SQ perspectives is essential for a more thorough understanding of UPA. However, the improvement in model performance was modest, implying that conflicts may exist between MBE and SQ features when interpreting UPA.

### Policy and design implications

Urban planning policies should consider the potential implications of integrating public transport with elements of cycling and pedestrian infrastructure to enhance park accessibility through multimodal approaches. Given the potential competition between bus and cycling trips, planners ought to contemplate establishing bicycle parks or shared bicycle services near bus stops. This proposal aligns with the objectives of the park and ride (P&R) policy^62^. This would encourage cycling for short trips to parks. Additionally, the positive impact of the proximity of metro stations on park accessibility in walking situations highlights the need to optimise the walking environment around metro stations^63^. Implementing wider pavements and safe zebra crossings, reducing waiting times at pedestrian overpasses and underpasses where motorised vehicles are still prioritised, and ensuring safety through adequate signage and maintenance, may further incentivise residents to walk to nearby parks. By synergising these approaches, cities can create a cohesive transport network that seamlessly integrates different modes of transport, promoting both sustainable mobility and increased park accessibility. From a street design perspective, increasing street network design may also be helpful for improved park accessibility. When creating new parks, the continuity and connectivity of the surrounding street network should be taken into consideration, and areas with a higher closeness centrality prioritised. In the case of existing parks, enhancing the connectivity and continuity of the adjacent roads may help to improve accessibility. The positive relationship between residential density and park accessibility that was found for walking mode, highlights the importance of proximity. It is thus recommended that municipalities contemplate developing and maintaining neighbourhood parks or mini parks, to address the challenges of insufficient urban park provision and their inequitable distribution^64^.

Overall, given the complementary nature of the macro-scale built environment and micro-scale streetscape features, urban planning and design professionals should shift their focus beyond conventional macro-scale considerations to include finer streetscape details within public spaces, where street-level interventions can be made in a noticeable and achievable manner^65,66^. Broad streets with greenery, pedestrian walkways, clear signage and memorable landmarks may facilitate easier park access. This is particularly important for active travel modes, especially walking, where the highest level of inequity was identified. Moreover, doing so offers a more economical alternative to constructing new parks or refurbishing old ones. It provides a cost-effective method for planners and policymakers to enhance park accessibility and lays the foundation for more equitable park access.

### Limitations and future research

This study has some limitations. First, it was designed with the basic concept of the ‘15-minute city’ in mind, and the service radius was defined accordingly. However, real-world park accessibility may extend beyond this framework. Future research could involve conducting a sensitivity analysis using different time frames (e.g. X-minute city) to better understand the impact of this uncertainty on accessibility and determine the most appropriate scale for measuring the effects of potential influencing factors. Second, this study assessed streetscape quality solely from an objective perspective, so future studies could explore the effects of both subjective and objective perceptions of these visual features on park accessibility in a comprehensive manner. Additionally, streetscape colour characteristics could be examined as another possible influencing factor. EDI considerations should include targeted groups, such as older adults, because individual characteristics influence people’s experiences of access to parks^9^. A more detailed examination of different park types, such as city-level and neighbourhood-level parks, could generate more precise policy implications to address inequitable access across various categories of parks. Lastly, looking beyond spatial and physical accessibility, a temporal perspective could offer insights into the availability of opportunities, transport service levels, and individuals’ time constraints^67^. Given the increase in valuable data and advanced models such as GeoAI, exploring park accessibility at a finer, spatio-temporal level using GeoAI would be a worthwhile direction for future research.

## Conclusions

This study has pioneered a GeoAI-based, multi-scale analytical framework in order to dissect the intricate dynamics between the built environment and urban park accessibility through various travel modes. By integrating advanced geospatial artificial intelligence with an in-depth examination of both macro- and micro-environmental factors, we have offered nuanced insights into how streetscape quality and the broader built environment shape park accessibility within a multimodal travel context.

This study contributes to the literature in two significant ways. First, it exposes disparities in urban park accessibility within the context of multimodal travel, enabling planners and policymakers to prioritise interventions by targeting travel modes with the greatest potential for addressing inequities through focused environmental justice and enhancements. Second, it integrates previously neglected micro-scale streetscape features into analyses of the role of the built environment in relation to park accessibility in a multimodal context, thus offering novel and valuable insights.

Our findings underscore the significant role of interconnected, broader, more open streets and more street-level greenery in facilitating access to urban parks, highlighting the potential for these elements to mitigate accessibility disparities across different travel modes. Moreover, the research elucidates the complementary relationship between macro-level elements of urban planning and the micro-level streetscape quality, emphasising the importance of a holistic, multi-scale approach to urban planning. Additionally, the findings offer a more economical alternative: improving streetscape quality could serve as a cost-effective method for authorities to enhance park accessibility, thereby eventually improving the equity of access to parks for residents.

In conclusion, the application of GeoAI in analysing park accessibility represents a significant step forward in urban planning research, offering a comprehensive perspective that bridges gaps in traditional studies. As cities evolve, this study contributes a valuable blueprint for enhancing the accessibility of urban parks - a key factor in promoting social equity, public health, and the well-being of city dwellers across the globe.

## Electronic supplementary material

Below is the link to the electronic supplementary material.

Supplementary Material 1

## Data Availability

The datasets used and/or analysed in this study are available upon reasonable request. For access, please contact Kaihan Zhang.
