# Supplementary material for: Illuminating the path to more equitable access to urban parks
Source: Sci Rep. 2025 Mar 20;15:9646. doi: 10.1038/s41598-025-94110-1 (PMC11926098; doi:10.1038/s41598-025-94110-1)
Supplement: Supplementary file 1 — Supplementary Material 1 [file 41598_2025_94110_MOESM1_ESM.docx]

**Illuminating the Path to more Equitable Access to Urban Parks**

**Supplementary Material**

| **Table S1 (Key definitions, formulae and explanations)** | | |
| --- | --- | --- |
| Variables | Explanation | Formulas |
| Greenness | Greenness refers to the presence of green elements within the streetscape, measured by the proportion of vertical vegetation or trees and other horizontal green features (plants, grass) in images. $PT PP$ denotes the total number of trees and terrains respectively. | $G=\frac{PT+PP}{P_{total}}$ |
| Openness | Openness refers to the perceived spatial expansiveness of a streetscape, quantified by the ratio of sky pixels to total pixels in an image. This measure affects pedestrian visibility and spatial experience. $PS_{k}$ denotes the total number of sky pixels in one image. | $O=\frac{PS_{k}}{P_{total}}$ |
| Enclosure | Enclosure refers to the height-to-width ratio of vertical structures (e.g., buildings, trees) in relation to horizontal elements (e.g., pavements, roads). $PB,PR, PS_{d}, PF$ denotes the total number of buildings, roads, pavements and fences respectively. | $E=\frac{\frac{PB}{P_{total}}+\frac{PT}{P_{total}}}{\frac{PR}{P_{total}}+\frac{PS_{\text{d}}}{P_{total}}+\frac{PF}{P_{total}}}$ |
| Walkability | Walkability refers to the relative ratio of pavements or sidewalks and fences to the total road surface, highlighting the visual impact of the horizontal perceptual environment on pedestrian experiences ^3^. $PS_{d}, PF, PR$ represent the total number of pavements, fences, and road pixels, respectively. Fences fulfil the function of separating roads from pavements, thereby enhancing pedestrian safety. | $W=\frac{\frac{PS_{d}}{P_{total}}+\frac{PF}{P_{total}}}{\frac{PR}{P_{total}}}$ |
| Imageability | Imageability refers to the characteristic of a physical setting that evokes a vivid mental image in viewers. It encompasses the qualitative value of an environment, often reflected in its diversity and visual richness, including distinctive buildings, signage, or other landmarks ^4^. $PB;PS_{sign}$ represent the total number of building and signboard pixels, respectively. | $I=\frac{PB}{P_{total}}+\frac{PS_{sign}}{P_{total}}$ |
| Streetscape Quality | Streetscape quality refers to the visual and functional characteristics of a street, encompassing all elements that are visible, experiential, and usable by individuals navigating or occupying the space. In this study, we assess streetscape quality through five distinct features, each capturing different dimensions of urban street experiences: Openness, Imageability, Walkability, Enclosure, and Greenness. | N.A. |

Note:$P=\sum_{i=1}^{4} V_{i}$, where $P$ represents the total number of pixels in each visual component, while $V_{i}$ signifies the number of pixels in visual components in a single directional image. *$P_{total}$* represents the total number of pixels for each sample point.

PSPNET ^1^ is designed for scene parsing and image segmentation. It captures multi-scale contextual information to enhance segmentation accuracy and semantic understanding. Traditional CNNs struggle with limited receptive fields, missing both the local and global context ^2^, which led to the development of PSPNET. Using pyramid structures, PSPNET extracts features at multiple scales for improved contextual modelling.

Its core components are a feature extraction network and a pyramid pooling module. To address challenges such as vanishing gradients and the curse of dimensionality, PSPNET utilises the pre-trained RESNET101, which efficiently captures both low- and high-level features from images.

**Text S2 (Urban park accessibility measurement)**

Urban park accessibility was quantified using the enhanced Gaussian-based 2SFCA method, which combined the Gaussian function and the original 2SFCA. To ensure the accuracy of the results and reflect the actual travel demand, this study considered three travel modes: walking, cycling, and driving. The specific calculation process was as follows:

The first step involved calculating the supply-demand ratio between parks and the population in each study unit. Each park can be regarded as a supply area: its area represents its service capability; the number of residents within a certain time limit represents the potential demand for the park; and its supply-demand ratio represents the balance or imbalance between the park's service capacity and the needs of the local population. The concept of a "living community" has recently attracted significant attention, with the "15-minute life circle" becoming a widely desired lifestyle. The 15-minute community living circle is fundamental in shaping the structure of community life. Consequently, this study adopted 15 minutes as the critical time threshold. Taking into account the actual road conditions in Guangzhou and the literature review, the average walking and cycling speeds were set at 5km/h and 15km/h respectively ^5^. In accordance with China’s highway engineering technical standards ^6^, driving speed limits were designated for different types of roads and vehicles. Accordingly, these speed constraints were used in this study. Driving speeds vary according to the category of the road: motorways have a speed limit of 100km/h;

national roads such as trunks roads have a speed limit of 80km/h; primary and secondary roads have speed limits of 60km/h and 50km/h respectively; and other roads have 40km/h speed limits.

This study identified all demand locations denoted as ‘$k$', characterised by their population grids, situated within a travel duration of '$t_{0}$' (threshold time) from each urban park entrance, denoted as '$j$'. We employed a Gaussian function (G) to assign weights to the population at each location '$k$'. By aggregating these weighted populations within each catchment area ($t_{kj}\leq t_{0}$), the potential demand can be determined. The supply-to-demand ratio ($R_{j}$) is:
$\begin{matrix} R_{j}=\frac{\text{S}j}{\sum k\in\{t_{kj}\leq t_{0}\}G(t_{kj},t_{0})P_{k}} \\ \end{matrix}$ (1)

$P_{k}$ is the population at location $k$ within the catchment area from urban park location $j$ with travel time $t_{kj}\leq t_{0}$. Here, $t_{kj}$is the transit time from $k$to $j$, S*j* is the park service capacity at$j$, and G is the time distance friction, as shown in the subsequent equation:

$G(t_{kj},t_{0})=\left\{\begin{matrix} \frac{e^{-(1/2)\times(t_{kj}/t_{0})^{2}}-e^{-(\frac{1}{2})}}{1-e^{-(\frac{1}{2})}},if,t_{kj}\leq t_{0} \\ 0\text{ },if,t_{kj}>t_{0} \end{matrix}\right.$ (2)

For every population grid unit '$k$', this study systematically investigated each urban park '$j$' within time '$t_{0}$'. Each $R_{j}$ value was adjusted using the time distance friction, and the resultant '$R$' values within each spatial unit were summed to calculate the spatial accessibility, represented as '$A_{k}$'.

$A_{k}=\sum_{j\in\{t_{kj}\leq t_{0}\}} G(t_{kj},t_{0})R_{j}$ (3)

**Table S3 (Robust LM test results and Moran’s *I* values by travel modes)**

|  | Walking mode | | | Cycling mode | | | Driving mode | | |
| --- | --- | --- | --- | --- | --- | --- | --- | --- | --- |
| Indicators | Coef. | Sig. |  | Coef. | Sig. |  | Coef. | Sig. |  |
| OLS Moran’s *I* on residuals | 0.864 | *** |  | 0.746 | *** |  | 0.682 | *** |  |
| Robust LM (lag) | 139.515 | *** |  | 12.915 | *** |  | 148.286 | *** |  |
| Robust LM (error) | 1728.649 | *** |  | 1096.185 | *** |  | 5082.971 | *** |  |
| Note:***,and ** signifies p < 0.01 and p < 0.05 respectively | | | | | | | | | |

**Text S4 (Street network closeness and betweenness calculation)**

This study employed an sDNA model to quantitatively evaluate the design metrics of street networks. Drawing upon spatial syntax theory, we selected 'closeness' and 'betweenness' as primary measurement metrics. Closeness (NQPD) quantifies street reachability and connectivity, typically calculated as the mean inverse distance from a specific link or node to all other links or nodes within a set radius. These metrics measure street connectivity, meaning that streets with higher closeness scores have shorter average distances to other streets in the network. The concept of betweenness captures whether people pass by a given location while moving within the network. A higher degree of betweenness suggests an increased likelihood of transit in an area, indicating that such streets commonly link major city areas or key locations, thereby reflecting the street network's overall functionality and efficiency.

We used $'n'$ to denote the calculation of the street network across the entire research area. A high closeness value indicates superior accessibility and the central positioning of a given area, as described by Equation (4):

$NQPDA(x)=\sum_{y\in R_{x}} \frac{p(y)}{d(x,y)}$ (4)

The Two-Phase Betweenness (TPBt) represents a mechanism whereby destinations compete against each other to attract origins. The TPBt measures the total flow directed towards a specific destination, thus accounting for the previously mentioned competition. The TPBt for a given street link, denoted as (x), was calculated using total geodesic standardisation within a defined radius, as outlined in the following equations.

$\text{OD}(\text{y},\text{z},\text{x})\text{=}\left\{\begin{matrix} 1, & \\ \text{x lying on the shortest path y to z} & \\ \text{1/2}, \text{x}\equiv\text{y}\neq\text{z} & \\ & \\ \text{1/2}, \text{x}\neq\text{y}\equiv\text{z} & \\ & \\ \text{1/3}, \text{x}\equiv\text{y}\equiv\text{z} & \\ & \\ 0, \text{othersituations} & \end{matrix}\right.$ (5)

$TPB_{t}(x)=\sum_{y\in N} \sum_{z\in Ry} OD(y,z,x)\frac{P(z)}{Links(y)}$ (6)

$OD(y,z,x)$ represents the minimal topological path from nodes $y$ to $z$ via node $x$ within radius $R$; $Links(y)$ denotes the total nodes within radius $R$ for node $y$; $P(z)$ indicates the weight of node $z$. The results are shown below:

| 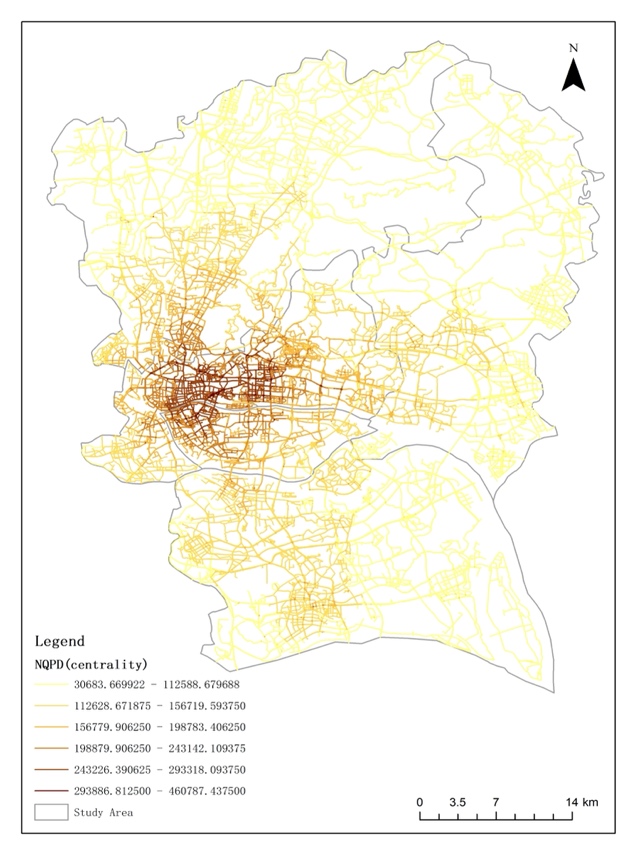 | 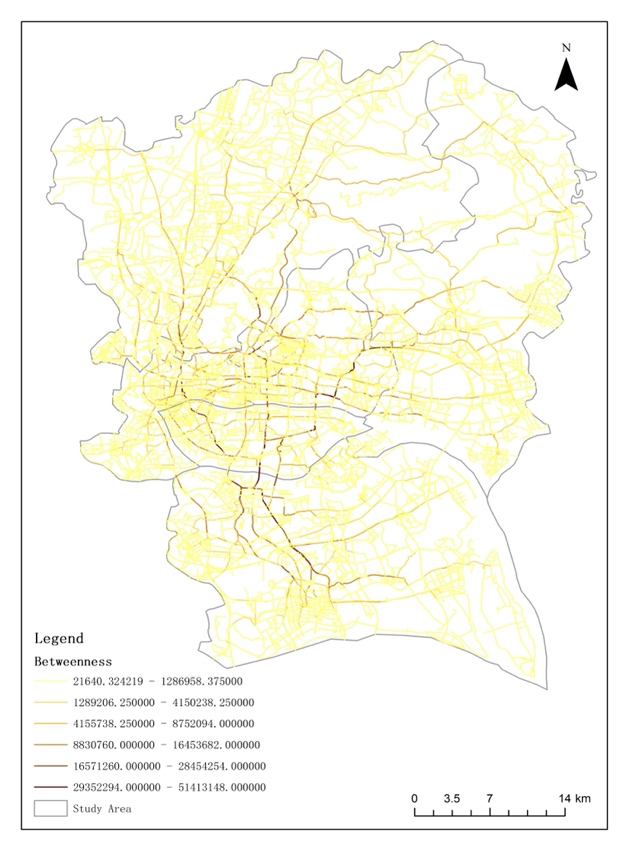 |
| --- | --- |

**References for Supplementary Material**

1. Zhao, H., Shi, J., Qi, X., Wang, X. & Jia, J. Pyramid Scene Parsing Network. in *IEEE Conference on Computer Vision and Pattern Recognition (CVPR)* (2017).

2. Sun, H. *et al.* A Spatial Analysis of Urban Streets under Deep Learning Based on Street View Imagery: Quantifying Perceptual and Elemental Perceptual Relationships. *Sustainability,* **15**, 14798 (2023).

3. Wang, X. *et al.* Adolescents’ environmental perceptions mediate associations between streetscape environments and active school travel. *Transportation Research Part D: Transport and Environment,* **114**, 103549 (2023).

4. Ma, X. *et al.* Measuring human perceptions of streetscapes to better inform urban renewal: A perspective of scene semantic parsing. *Cities,* **110**, 103086 (2021).

5. Wang, J., Kwan, M-P., Liu, D. & Peng, X. Assessing the spatial distribution of and inequality in 15-minute PCR test site accessibility in Beijing and Guangzhou, China. *Applied Geography,* **154**, 102925 (2023).

6. JTG B01-2014. Technical standard of highway engineering. (2014).
